# Effect of Dexmedetomidine on Levobupivacaine and Ropivacaine in Fascia Iliaca Block for Trochanteric Fractures Treated by Proximal Femoral Nail – A Randomized Trial

**DOI:** 10.7759/cureus.5352

**Published:** 2019-08-09

**Authors:** Bhavna Sriramka, Sandeep K Panigrahi, Ranjita Acharya, Jayanti Singh

**Affiliations:** 1 Anesthesia, Institute of Medical Sciences and SUM Hospital, Bhubaneswar, IND; 2 Community Medicine, Institute of Medical Sciences and SUM Hospital, Bhubaneswar, IND; 3 Anestheisa, Institute of Medical Sciences and SUM Hospital, Bhubaneswar, IND

**Keywords:** dexmedetomidine, levobupivacaine, ropivacaine, local anaesthetics, analgesia, fascia iliaca block, femoral fractures, pain management

## Abstract

Background: Fascia iliaca block (FIB) is an established procedure for postoperative pain relief in femur fracture surgeries. Dexmedetomidine was found to be a useful additive to local anesthetics (LA) for peripheral nerve blocks resulting in a prolonged anesthetic effect. We evaluated the impact of the addition of dexmedetomidine to an equal concentration of levobupivacaine and ropivacaine for FIB.

Methods: The present study is a double-blind, randomized trial conducted on 60 patients in the age group 18-70 years. The patients were divided into two groups: Group LD received 30 ml of an LA solution (29.5 ml 0.25% levobupivacaine and 0.5 ml dexmedetomidine 50 µg), and group RD received 30 ml of another LA solution (29.5 ml 0.25% ropivacaine and 0.5 ml dexmedetomidine 50 µg). The outcomes observed were the duration of analgesia (DOA) and total analgesic requirement (TAR).

Results: The DOA was found to be slightly longer in group LD (955.3 ± 114.5 minutes) than in group RD patients (894.6 ± 91.3) with p = 0.027. The TAR was found to be statistically different though clinically irrelevant (group LD: 112 mg; IQR: 105-122 vs. Group RD: 115 mg; IQR: 104-118, p = 0.034, where IQR stands for interquartile range). There were no signs of neurotoxicity in any of the participating patients.

Conclusion: Addition of 50 µg dexmedetomidine to 0.25% levobupivacaine extends DOA slightly as compared to when dexmedetomidine is added to 0.25% ropivacaine. However, TAR remains clinically the same for both cases in fascia iliaca block.

## Introduction

Regional blocks are an essential tool in the field of pain management [1]. Ultrasonographic guidance has made the technique safer and reduced systemic side effects [2]. Fascia iliaca block (FIB) has proved of immense value for patient positioning, especially for patients having a femur fracture [2]. In the preoperative room, FIB acts by relieving the patient’s anxiety and reducing the anesthetic requirement. Postoperative analgesia is also reduced by the pre-emptive action of FIB on surgical incision related pain [3]. Successful pain management enhances early ambulation and reduces hospital stay [4]. Researchers have been trying a variety of drugs and their combinations in FIB [5]. Local anesthesia (LA) drugs such as bupivacaine, levobupivacaine, and ropivacaine are the most commonly used analgesics for this block. Being a pure sensory block, a lower concentration of LA, i.e., 0.25%, has been found to be effective [5]. Numerous adjuvants (opioid, dexamethasone, α-adrenergic agonists such as clonidine, dexmedetomidine) are being tested to enhance the duration and reduce the onset time of blocks [6].

Levobupivacaine and ropivacaine are pure S(-) isomers of the family of n-alkyl-substituted pipecholyl xylidines that are preferred because of their clinical safety. Although their physicochemical properties are quite similar, their clinical results differ [4]. A recent meta-analysis suggests that the onset time of the FIB block was similar for both the anesthetics [7]. Studies with dexmedetomidine as an additive to either levobupivacaine or ropivacaine have shown that the adjuvant prolongs the duration of the analgesia in axillary and brachial plexus blocks [8,9]. However, there is no comparative data on the improvement in duration of analgesia (DOA) and total analgesic requirement (TAR) when dexmedetomidine is added to levobupivacaine and ropivacaine for a pure sensory block such as FIB.

The authors aim to study this drug potentiating effect of dexmedetomidine on levobupivacaine and ropivacaine, both held at equal concentrations. They hypothesize that the addition of dexmedetomidine will have a synergistic effect on ropivacaine and levobupivacaine. The primary objective of the study is to assess the duration of postoperative analgesia; the secondary goal is to measure the total analgesic requirement, determine the hemodynamic changes that may have occurred and look for any side effects or complications.

## Materials and methods

The study was conducted in a tertiary hospital located in eastern India between November 2018 and April 2019 after approval by the institute’s Research and Ethics Committee and registration on the ClinicalTrials.gov.in (CTRI/2018/12/016670) site. A written and signed informed consent form was obtained from all the recruited patients after being given full information about the procedure. Data were collected from the preoperative room, operation theatre, and postoperative indoor orthopedic department.

The present study was a parallel-design non-inferiority randomized control trial. It involved two arms (LD and RD) with an allocation ratio of one. One group, group LD, received FIB 29.5 ml of levobupivacaine (Levo-Anawin; Neon laboratories, Mumbai, India) 0.25% with 0.5 ml (50 µg) of dexmedetomidine (Dexem; Themis Medicare, Uttarakhand, India) and the other group, group RD, received FIB 29.5 ml of ropivacaine (Ropin; Neon laboratories, Mumbai, India) 0.25% with 0.5 ml (50 µg) of dexmedetomidine to make a total volume of 30 ml. Adult patients (18-70 years) with grade I or II (as classified by the American Society of Anesthesiologists) trochanteric femur fractures who had undergone proximal femoral nailing (PFN), performed by surgeons with similar surgical expertise, under spinal anesthesia were incorporated in the study. Patients with known hypersensitivity to any of the drugs under study (dexmedetomidine, levobupivacaine or ropivacaine); with deranged coagulation profile; on medication such as opioids or adrenoreceptor antagonists/agonists; poly-trauma; past history of severe cardiac, respiratory, renal or hepatic disease; pregnancy and those changed from spinal to general anaesthesia were omitted from study. Similarly, patients having local site infection or altered anatomy like inguinal hernia, and femoral artery graft were also excluded. Trochanteric femoral fractures requiring implants other than PFN were not included in this study. Finally, patients were only included if they were able to position comfortably for spinal anesthesia (reasonable analgesia) post block.

The sample size was calculated using an online sample size calculator available from Harvard University [10]. Using a two-sided test with a power of 85 percent and a significance level of 0.05 and assuming that the mean duration of analgesia of one group is located at the 80th percentile of the other, it was estimated that a sample size of 54 patients was needed for this two-treatment parallel-design study. Considering a dropout (due to either block failure or conversion to general anesthesia) of 10%, a minimum of 60 subjects was considered necessary for the study. The investigator cum clinical trial coordinator (SP) performed randomization using a computer-generated random number series. He also ensured allocation concealment through sealed, opaque envelopes. Patient enrolment was done by either of the anaesthesiologists (BS, JS). Assignment of participants to a particular intervention was done by SP. Double blinding was ensured as the participants, interventionist and the observer of the outcomes were unaware of group allocation of the participants.

In the preoperative room, intravenous access was established and monitors were connected. Intravenous midazolam (1 mg) was administered for anxiolysis before the block was conducted. The block was executed by an experienced anesthesiologist (BS, having performed more than 100 ultrasonography-guided FIB) blinded to the randomization process and drug preparation. Ultrasound (Sonosite M Turbo, Washington, USA) was used for administration of the block. The skin was prepared with disinfectant, and a transducer probe was used to localize the neuro-vascular-muscular structures and fascia iliaca. The probe was then shifted laterally to identify the sartorius. The needle was introduced through the fascia iliaca using the in-plane technique as the fascia snapped back on the ultrasound image. After negative aspiration, 30 ml of the prepared drug solution was instilled and the separation of the fascia iliaca was noted. The depth of the block was assessed after 20 minutes by appraising the comfortability of the patient for positioning for spinal anesthesia (SA). Heavy bupivacaine 0.5% (15 mg) was used as a standard dose for SA in all patients. Three surgeons with similar skills performed the proximal femur nail (PFN) surgeries. Tablet paracetamol 500 mg was given to all patients at a twice-daily dosage for supplemental analgesia. Patients received intravenous 100 mg of tramadol as “rescue analgesia” for “breakthrough pain” [visual analogue scale score (VAS) > 3].

The primary outcome was evaluated by noting the duration of analgesia, i.e., the time interval between block administrations and the first time the patient feels pain at an intensity of VAS > 3. The secondary outcome was measured as the entire dose of tramadol consumed by the patient 48 hours post FIB. Patients were also monitored for any untoward impact of dexmedetomidine like hypotension or bradycardia or any neurotoxic adverse effects like paresthesia, altered motor movements and heightened sensitivity to temperature changes. Long-term complications like difficulty in walking, however, could not be assessed.

Data were entered into an excel sheet and cleaned. They were then imported into IBM SPSS v 20.0 licensed to the university. Continuous data were expressed as mean with standard error (for normal distribution) and median with interquartile range (for non-normal distribution). Normality check was done using a Kolmogorov-Smirnov test/Shapiro-Wilk test. Comparison between the groups was made at a = 0.05 levels using the appropriate statistical test (Mann-Whitney U test or an independent t-test depending upon the normal distribution of the data). Tables and graphs were used to depict the findings.

## Results

**Figure 1:**
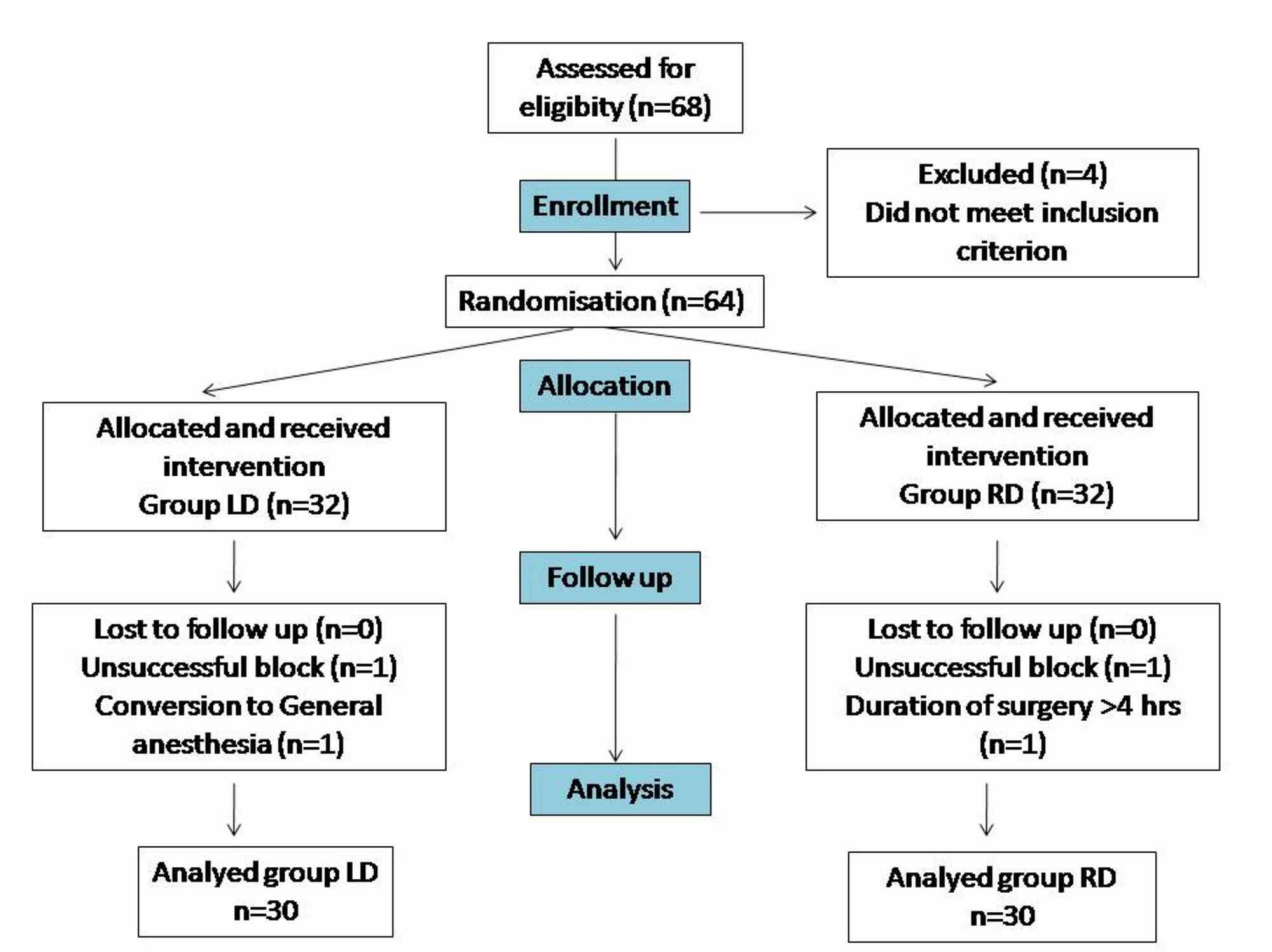
Consolidated Standards of Reporting Trials (CONSORT) flow diagram of participants

Sixty-eight patients were willing to participate in the study. Out of these, four were excluded in the initial phase (n = 4 did not meet the inclusion criteria). The rest (n = 64) were randomized and allocated to the two different intervention groups (groups LD and RD), with proper allocation concealment in place (n = 32 each). The interventions were applied to the patients and followed up. There was no loss to follow up in either group (n = 0). However, some of them were excluded from the study due to unsuccessful blocks, changes in surgical plans or surgical time exceeding four hours in both groups (n = 4). Thus, 60 patients were analyzed (n = 30 in each group). Details have been summarized using the CONSORT (Consolidated Standards of Reporting Trials) flow diagram (Figure 1).

The difference in the mean age between the groups was not found to be statistically significant (p = 0.224). Similarly, the groups were also comparable with regard to gender (p = 0.243) and duration of surgery (p = 0.673). This ensured that randomization was correctly done, and the two groups were similar in their baseline characters (Table 1).

**Table 1:** Baseline parameters comparison between the two groups (n=30 each)

		Group LD	Group RD
Age of the patient (in years)	Mean	48.60	45.10
	SD	11.65	10.34
Male Gender	Numbers	24	20
	Percent	80%	66.7%
Female Gender	Numbers	6	10
	Percent	20%	33.3%
Duration of surgery (in minutes)	Median	112.0	115.0
	IQR (Q_1_-Q_3_)	(105,122)	(104,118)

Mean DOA was found to be a little longer in group LD (955.3 ± 114.5 minutes) as compared to group RD (894.6 ± 91.3), and this was found to be statistically significant (p = 0.027) (Table 2).

**Table 2:** Duration of analgesia and requirement of tramadol for the study participants in the two study groups (n = 30 each) 1 Independent Sample t-test; 2 Mann-Whitney U-test;* Significant at a = 0.05 levels

		Group LD	Group RD	P value
Duration of analgesia (in minutes)^1^	Mean	955.30	894.60	0.027*
	SD	114.48	91.27	
Requirement of tramadol (in mg)^2^	Median	112.0	115.0	0.034*
	IQR (Q_1_-Q_3_)	(105,122)	(104,118)	

**Figure 2:**
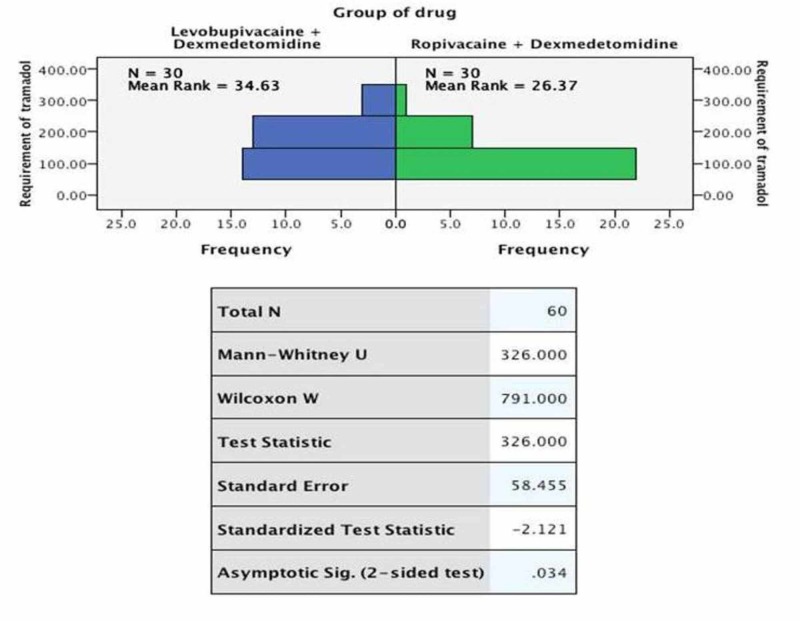
Comparison of requirement of tramadol between the study groups (n = 30)

The TAR was also found to be statistically different though clinically irrelevant (group LD: 112 mg; IQR: 105, 122 vs. group RD: 115 mg; IQR: 104, 118, p = 0.034). No patient showed any sign of neurotoxicity (Figure 2).

## Discussion

Fractures around the hip are common both in the elderly, owing to osteoporosis, and in the young due to road traffic accidents [11]. Effective pain management improves the long-term quality of life [12]. FIB is an integral part of the multimodal analgesia that is used to provide postoperative analgesia in lower limb orthopedic surgeries as well as to burns patients [6,13]. This single intervention blocks four nerves, namely, the femoral, lateral femoral cutaneous nerve, obturator and the genitofemoral. However, FIB is more of a sensory block. Ultrasound guidance has improved the accuracy of anesthetic injections and increased procedural safety [14]. Anesthetists have contended with the fact that adjuvants to LA in FIB prolong postoperative analgesia and reduce the requirement of drugs, particularly opioids that have detrimental effects on body systems [2].

Since the emergence of reports regarding bupivacaine-related severe toxicity, several newer long-acting LAs have been introduced in clinical practice, of which levobupivacaine and ropivacaine are the most studied. Both being pure left-isomers, they have less cardiac and neural toxicity [15]. The LAs differ slightly in terms of anesthetic potency, with racemic bupivacaine > levobupivacaine > ropivacaine, but the properties of these three drugs are very similar. However, the substitution of the pipecoloxylidine with a 3-carbon side-chain instead of a 4-carbon side-chain in the ropivacaine molecule makes it less lipophilic than the other two. Some studies found levobupivacaine to be apparently more potent than the other drugs. It was hypothesized that as ropivacaine concentrations were presented as hydrochloride salts rather than bases like levobupivacaine, their strength was underestimated by 13% [16]. Ropivacaine has a more selective impact on nociceptive (Aδ and C) fibers than on motor fibers, which give rise to a faster onset of sensory block [17].

Animal studies by Brummett et al. showed that dexmedetomidine enhances the duration of anesthesia and analgesia when used with bupivacaine or ropivacaine during sciatic nerve block in rats. The mechanism is via the hyperpolarization-activated cationic channel [18,19]. In human studies, a dexmedetomidine-lidocaine mixture has been used to provide Bier’s block and was shown to improve the quality of anesthesia, decrease tourniquet pain and reduce postoperative analgesic requirement [20]. Helal et al. also demonstrated that the addition of dexmedetomidine to bupivacaine in a combined sciatic-femoral nerve block improved the analgesic effect but found significant bradycardia and hypotension in their patients requiring treatment [21]. Most authors have similarly reported (using 100 µg dexmedetomidine) side effects of dexmedetomidine such as hypertension, hypotension, bradycardia or hyperglycemia [22,23]. Rashmi et al. used 50 µg dexmedetomidine instead of 100 µg and found that though there was a reduction in heart rate and blood pressure, the drop was not significant enough to warrant specific treatment [24]. Swami et al. also found in their study that a group treated with 1 µg/kg dexmedetomidine added to LA had lower heart rate (HR) and blood pressure (BP) as compared to a group treated with 1 µg/kg clonidine added to LA [25]. However, these drops were non-significant. To use a safe dose of dexmedetomidine, we decided to operate with 50 µg dexmedetomidine in both our groups as practically it is difficult in patients having fracture femur to calculate weight and dosage on per kg body weight. We noted a fall in HR and BP from the baseline, but these were not alarming and common to both groups. Hence, we excluded this as a measurable outcome in our study. The choice of the lower dose was reinforced by the choice of our block. FIB, being a predominantly sensory block, has minimal motor effects; a lower dose with lesser systemic resorption and minor side effects would be ideal for our situation. Swami et al. also found that dexmedetomidine produced a faster onset of action as compared to clonidine. In a sensory nerve block such as FIB, this has significance [25].

Gupta et al. studied two groups treated with 0.25% of levobupivacaine and ropivacaine in fascia iliaca block. They found that the DOA is similar in both the groups and the difference is statistically insignificant (572.0 ± 269.2 vs. 534.55 ± 166.90 minutes). However, they did not study the total consumption of analgesic in the two groups [5]. In our groups, the DOA is found to be longer as we added dexmedetomidine. In group LD, it was 955.3 ± 114.5 minutes and in group RD, it was 894.6 ± 91.3 minutes. This shows the synergism or additive nature of dexmedetomidine. A study by Kumar et al. on femoral nerve block for femur fracture surgeries found ropivacaine with dexmedetomidine better than ropivacaine alone in terms of onset of action (3.77 ± 0.84 min vs. 4.6 ± 1.1 min), DOA ( 744.33 ± 179.6 min vs. 263 ± 67 min) and analgesic consumption (2 ± 0.06 vs. 3.3 ± 0.6) [26]. However, Li et al. studied the same in FIB, and they concluded that ropivacaine alone or with dexmedetomidine were similar in terms of the DOA but with lesser rescue analgesic requirement in the latter group [7]. The difference in results may be attributed to a smaller sample size by Li et al. (n = 20 in each group). In another study, Biswas et al. compared levobupivacaine alone, with dexmedetomidine being used as an additive in a supraclavicular block. They found the combination was superior in terms of DOA and requirement of rescue analgesia [27].

We acknowledge that we have certain limitations in our study. We have used a fixed 50 µg dosage of dexmedetomidine based on literature review and difficulty to calculate dosage based on measurement per weight. The alterations in the hemodynamic parameters with the addition of dexmedetomidine were not studied. A comparative trial with LA (levobupivacaine, ropivacaine) alone and with dexmedetomidine would have better delineated the subtle hemodynamic variation. The VAS scores at time intervals were not recorded as only the rescue analgesia was provided whenever there was breakthrough pain (VAS > 3). Thus, the quality of analgesia was not studied nor was sedation or patients prospective in terms of satisfaction interviewed. Quadriceps weakness was difficult to assess in the elderly patients as they were not mobilized following day as per orthopedic protocol and quadriceps-tightening exercises were subjective. Another shortcoming was the lack of follow up to check for any long-term neurological complications.

## Conclusions

In conclusion, FIB provides adequate analgesia for the positioning of patients for spinal anesthesia in trochanteric femoral fractures. Dexmedetomidine 50 µg when added to levobupivacaine fares slightly better in terms of duration of analgesia as compared to dexmedetomidine 50 µg when added to ropivacaine. However, the total analgesic requirement remains clinically similar.
